# Genetic Risk for Psychiatric Disorders and Telomere Length

**DOI:** 10.3389/fgene.2018.00468

**Published:** 2018-10-16

**Authors:** Alish B. Palmos, Gerome Breen, Laura Goodwin, Souci Frissa, Stephani L. Hatch, Matthew Hotopf, Sandrine Thuret, Cathryn M. Lewis, Timothy R. Powell

**Affiliations:** ^1^Social, Genetic and Developmental Psychiatry Centre, Institute of Psychiatry, Psychology and Neuroscience, King’s College London, London, United Kingdom; ^2^National Institute for Health Research Biomedical Research Centre for Mental Health, Institute of Psychiatry, Psychology and Neuroscience, Maudsley Hospital, King’s College London, London, United Kingdom; ^3^Department of Psychological Medicine, Institute of Psychiatry, Psychology & Neuroscience, King’s College London, London, United Kingdom; ^4^Department of Psychological Sciences, University of Liverpool, Liverpool, United Kingdom; ^5^Health Service & Population Research Department, Institute of Psychiatry, Psychology & Neuroscience, King’s College London, London, United Kingdom; ^6^South London and Maudsley NHS Foundation Trust, London, United Kingdom; ^7^Department of Basic and Clinical Neuroscience, Institute of Psychiatry, Psychology and Neuroscience, King’s College London, London, United Kingdom

**Keywords:** polygenic risk score, psychiatry, antidepressants, aging, telomeres

## Abstract

**Background:** Previous studies have revealed associations between psychiatric disorder diagnosis and shorter telomere length. Here, we attempt to discern whether genetic risk for psychiatric disorders, or use of pharmacological treatments (i.e., antidepressants), predict shorter telomere length and risk for aging-related disease in a United Kingdom population sample.

**Methods:** DNA samples from blood were available from 351 participants who were recruited as part of the South East London Community Health (SELCoH) Study, and for which whole-genome genotype data was available. Leukocyte telomere length was characterized using quantitative polymerase chain reactions. Individualized polygenic risk scores for major depressive disorder (MDD), bipolar disorder (BD), and schizophrenia (SCZ) were calculated using Psychiatric Genomics Consortium summary statistics. We subsequently performed linear models, to discern the impact polygenic risk for psychiatric disorders (an etiological risk factor) and antidepressant use (common pharmacological treatment) have on telomere length, whilst accounting for other lifestyle/health factors (e.g., BMI, smoking).

**Results:** There were no significant associations between polygenic risk for any of the psychiatric disorders tested and telomere length (*p* > 0.05). Antidepressant use was significantly associated with shorter telomere length and this was independent from a depression diagnosis or current depression severity (*p* ≤ 0.01). Antidepressant use was also associated with a significantly higher risk of aging-related disease, which was independent from depression diagnosis (*p* ≤ 0.05).

**Conclusion:** Genetic risk for psychiatric disorders is not associated with shorter telomere length. Further studies are now needed to prospectively characterize if antidepressant use increases risk for aging-related disease and telomere shortening, or whether people who age faster and have aging-related diseases are just more likely to be prescribed antidepressants.

## Introduction

The complex and dynamic relationship between physical illness and psychiatric disorders was highlighted in a Chief Medical Officer’s 2013 annual report, which stated that people with a psychiatric disorder experience worse physical health than those without (Davies, 2014). The comorbidity of a long-term physical illness and psychiatric disorder raises total health care costs by at least 45% per person (Naylor et al., 2012), and increases the risk of early mortality (Chang et al., 2011).

Psychiatric disorders such as major depressive disorder (MDD), bipolar disorder (BD), and schizophrenia (SCZ) have all been linked to an increased risk of severe medical conditions throughout a person’s life (Kessler et al., 2005; Rai et al., 2014; Kang et al., 2015; Menear et al., 2015). The prevalence of MDD in patients with a physical illness is reported to be around twofold to threefold higher than in the general population and comorbid illnesses such as diabetes, pain, cancer, stroke, and cardiovascular disease have become an increasingly important global health issue (Kessler et al., 2005; Kang et al., 2015; Winkler et al., 2015). A cross-sectional report on patients with schizophrenia has stated that more than 50% of patients with schizophrenia possess at least one comorbid physical illness such as chronic pain, liver disease, and in particular, type-2 diabetes, which can often lead to microvascular and macrovascular complications such as neuropathy, coronary heart disease, and stroke (Chwastiak et al., 2006; Meeuwisse-Pasterkamp et al., 2008; Smith et al., 2013). BD is also associated with medical conditions likely to increase mortality, including respiratory, cardiovascular, and endocrine problems (Kemp et al., 2010; Forty et al., 2014).

Interestingly, medical conditions that show the highest prevalence of comorbidity across these three psychiatric disorders (MDD, BD, and SCZ) tend to be associated with aging; namely cardiovascular disease, stroke, obesity, and type-2 diabetes. This suggests that psychiatric disorders may be associated with faster biological aging, and some studies which assay ‘telomeres’ support this notion (e.g., Simon et al., 2006).

Telomeres are found on the ends of chromosomes and are special structures that are essential for protecting the chromatin from DNA damage during recombination (de Lange, 2002). Telomeres get shorter with each cell division as a result of the end-replication problem and this progressive telomere shortening is thought to represent a ‘molecular clock’ which underlies cell aging (Blackburn, 2001; Collins and Mitchell, 2002). Telomere shortening to a critical length, results in the realization of the “Hayflick limit” and a reduction in the ability of cells to divide (Blackburn, 2001). Ultimately, this means that new cells are less able to replace old, damaged cells, and thus the body becomes more vulnerable to aging-related diseases (Blackburn, 2001; Samani et al., 2001; Collins and Mitchell, 2002; Oh et al., 2003; Blasco, 2005).

Shorter telomeres have been demonstrated in patients suffering from MDD, BD, and SCZ compared to controls, leading to speculation that telomeres may play a role in psychiatric disorder etiology (Simon et al., 2006; Lung et al., 2007; Kao et al., 2008; Yu et al., 2008; Hartmann et al., 2010; Elvsåshagen et al., 2011; Wikgren et al., 2012; Lima et al., 2015; Lindqvist et al., 2015; Mamdani et al., 2015). However, understanding whether or not telomere shortening is directly related to the pathophysiology of psychiatric disorders is difficult to determine from classic case-control studies due to confounding factors. Specifically, psychiatric patients recruited to case-control studies are often already taking medications and are significantly more likely to be leading unhealthy lifestyles (e.g., poor diet, smoking), which may impact upon rates of telomere shortening. Subsequently, what may be more informative is to study a known causative risk mechanism for a psychiatric disorders and its association with telomere length, outside the context of the disorder itself and associated environments - polygenic risk scores (PRS) represent one option which may allow us to achieve this.

Polygenic risk scores represent the cumulative effect of many common risk variants for a given trait and are an effective way of quantifying genetic risk for a psychiatric disorder, even in non-clinical population cohorts (Sullivan, 2010; Clarke et al., 2016). PRS have previously been used to better understand the effect genetic risk mechanisms have on biological systems and clinical symptoms (e.g., Alloza et al., 2017). A recent study from our group has shown the importance of studying genetically at-risk, but clinically unaffected individuals, when investigating telomere length differences (Powell et al., 2017a). We previously found that unaffected first-degree relatives of BD patients have shorter telomeres compared to control participants, implying an association between familial risk for BD and shorter telomeres. This effect was not clear amongst BD cases, potentially as a result of lithium use, which was associated with longer telomeres within BD patients. Subsequently, PRS allows us to draw similar comparisons in any given population, with genetic risk being quantified empirically using genome-wide genotype data, as opposed to familial relatedness.

In addition to lithium use, other more commonly used pharmacotherapies have been implicated in affecting telomere length (Lindqvist et al., 2015; Monroy-Jaramillo et al., 2017). Recent studies have revealed that only depressed patients taking antidepressants have significantly shorter telomere lengths relative to controls; with the reports surmising that the effect is likely due to the more severe nature of depression in those requiring medication (Lindqvist et al., 2015; Needham et al., 2015). However, epidemiological studies independently reveal associations between antidepressant use and an increased risk for aging-related disease, which is irrespective of depression diagnosis (Hippisley-Cox et al., 2001; Gareri et al., 2002; Caughey et al., 2010). Thus, there is warrant for further investigation on the effects of antidepressant medication on telomere length, especially when used outside the context of depression, as this would allow one to tease apart the impact of psychiatric diagnosis from the impacts of medication.

Our study comprised of 351 participants from a South East London, United Kingdom population cohort. We aimed to investigate whether: (i) PRS for MDD, BD, or SCZ predicts shorter relative telomere length in the non-clinical majority of the sample; (ii) antidepressants impact on telomere length, irrespective of depression diagnosis, and (iii) if any significant associations from (i) or (ii) are additionally associated with risk for aging-related disease.

## Materials and Methods

### Participants

A cohort study design was used to address the research questions. A total of 351 participants including 167 males (mean age of 50 (16.6 S.D.)) and 184 females (mean age of 47 (14.1 S.D.)) had their blood samples collected for DNA extraction as part of the South East London Community Health study (SELCoH; Hatch et al., 2011, 2016), see **Table 1**. Depression was the only common clinical psychiatric diagnosis in SELCoH, based on self-report data (*n* = 61). Current depression severity (at the time of blood collection) was coded as an ordinal measure (0 = no depression symptoms, 1 = mild depression symptoms, 2 = moderate-severe depression symptoms), using the Clinical Interview Schedule-Revised (CIS-R; Lewis et al., 1992), which uses an algorithm to approximate ICD-10 diagnoses (World Health Organisation [WHO], 1993).

**Table 1:** The characteristics of our sample at the time of blood collection, including gender, age, BMI, smoking status, current antidepressant use and average polygenic risk scores (PRS), in depressed cases (Dep+) and non-depressed controls (Dep-).

	Dep- sample	Dep+ sample
*n*	290	61
Age	48.43 (15.58)	48.36 (14.39)
Sex (% male)	142 (49)	25 (41)
BMI	27.04 (5.39)	27.99 (6.25)
Ethnicity	White British: 165	White British: 35
	Black Caribbean: 24	Black Caribbean: 6
	Black African: 33	Black African: 3
	White other: 39	White other: 12
	Non-white other: 18	Non-white other: 4
	Mixed: 11	Mixed: 1
Smoking (n)	Never: 122	Never: 14
	Current: 53	Current: 24
	Ex-smoker: 115	Ex-smoker: 23
Currently taking antidepressants (n)	10	30
PRS MDD	-0.0029	0.0137
PRS BD	-0.0141	0.067
PRS SCZ	-0.0164	0.078

To examine the relationship between PRS and telomere length, independent from the confounders of disease factors and medication in SELCoH, we split our sample into those without a reported depression diagnosis (dep-), and those with a reported depression diagnosis (dep+). DNA from participants was extracted from blood samples and this was used to calculate RTL and assay common genetic variation.

### Ethics Statement

The SELCoH study received approval from King’s College London research ethics committee, reference PNM/12/13-152. Informed written consent was obtained from all participants at the time of sample collection.

### Data Availability Statement

Due to ethical restrictions SELCoH data is not publically available. Details on the SELCoH sample and requests to access phenotype data can be made here: http://www.slam.nhs.uk/research/selcoh/selcoh-projects. Access to genetic data requires local approval via the NIHR Bioresource (contact: bioresource@kcl.ac.uk).

### Aging-Related Disease

We constructed an ordinal measure for aging-related disease, whereby 0 indicated no reported aging related disease, 1 indicated one reported aging-related disease, and 2 indicated two or more aging related diseases. Aging-related diseases included: type-2 diabetes, arthritis, cardiovascular disease, stroke, high blood pressure, and cancer.

### DNA Extraction and Telomere Assessment

10 mL of blood was collected from participants in tubes containing EDTA (BD Vacutainer; BD, NJ, United States) and stored at -80°C. DNA was then extracted using a standard in-house protocol (Freeman et al., 2003) and stored at -80°C. All samples had 260/280 ratios of between 1.7 and 1.9, tested using the Nanodrop D1000 (Thermoscientific, Wilmington, DE, United States).

To assess relative telomere length (RTL), we performed a modified version of a quantitative polymerase reaction (qPCR) protocol by Cawthon (2009), as previously described (Powell et al., 2017a; Vincent et al., 2017). The protocol involves two separate qPCRs performed on separate 384-well plates with DNA samples pipetted into identical wells on each plate. In the first reaction, we assayed the telomere repeat region (TTAGGG). In the second reaction, we assayed a single copy gene, albumin, which we used as an internal control to correct for differences in DNA concentration between samples (Cawthon, 2009). The telomere/albumin ratio was used to calculate RTL.

On each plate, six negative controls consisting of RNase-free water were used to screen for any DNA contamination. An eight-point dilution series using human leukocyte genomic DNA (0.47, 0.94, 1.88, 3.75, 7.5, 15, 30, and 60 ng) was used on each plate to allow for absolute quantification of each sample and to account for any differences in efficiency between the telomere and albumin reactions. All reactions were performed using three technical replicates. Each qPCR mix for the telomere reactions consisted of 10 μL of 2x qPCR Mastermix with SYBR Green (Primer Design, Southampton, United Kingdom), 5 μL or RNase free water, 12 ng of DNA, 1000 nM of telg, 5^′^-ACACTAAGGTTTGGGTTTGGGTTTGGGTTTGGGTTAGTGT-3^′^ and 800nM of telc, 5^′^-TGTTAGGTATCCCTATCCCTATCCCTATCCCTATCCCTAACA-3^′^. Four stages made up the thermocycling conditions as follows: Stage 1: 95°C for 15 min, Stage 2: 2 cycles for 15 s at 94°C and 49°C, Stage 3: 25 cycles at 94°C for 15 s, 10 s at 62°C, and 15 s at 73°C (data collection), Stage 4: dissociation curve (primer specificity detection).

The same reagents and quantities were used for the albumin reactions, apart from the albumin forward and reverse primers replaced the telomere primers. Quantities of the albumin forward and reverse primers were adjusted to 765 nM for the forward primer albu, 5^′^-CGGCGGCGGGCGGCGCGGGCTGGGCGGAAATGCTGCACAGAATCCTT-3^′^ and 930 nM for the reverse primer albd, 5^′^-GCCCGGCCCGCCGCGCCCGTCCCGCCGGAAAAGCATGGTCGCCTGTT-3^′^. The thermocycling conditions for the albumin reaction consisted of four stages: Stage 1: 95°C for 15 min, Stage 2: 2 cycles for 15 s at 94°C and 49°C, Stage 3: 33 cycles at 94°C for 15 s, 10 s at 62°C, and 15 s at 88°C (data collection), Stage 4: dissociation curve (primer specificity detection).

Reactions were performed using either the ABI Prism 7900HT Sequence Detection System (Thermofisher Scientific, MA, United States) or the QuantStudio 7 Flex Real-Time PCR System (Thermofisher Scientific).

### Genotyping and Quality Control (Target Dataset)

DNA samples were sent to the Affymetrix Research Services Laboratory in Santa Clara, California, CA, United States. Genotyping for SELCoH was assayed using the United Kingdom Biobank Axiom Array (r3) which comprises of 820,967 genetic markers (Affymetrix, California, CA, United States). Genotype data was put through quality control measures as outlined by Coleman et al. (2016), using PLINK v1.07 (Purcell et al., 2007) and mapped to genomic build hg19. Specifically, patient samples were excluded if there was greater than 5% missingness in genotype data, and individual SNPs were excluded if there was greater than 5% missingness. A minor allele frequency (MAF) threshold was set to 0.05, and a Hardy-Weinberg threshold of 0.00001, in keeping with what’s recommended for smaller sample sizes (Coleman et al., 2016).

The absence of sample mismatching was confirmed using sex checks, where genetic sex was compared to phenotypic sex. The genome-wide Identity by Descent (IBD) analysis which is performed between pairs of samples, measured the probable number of shared alleles at any given marker, and was used to identify and exclude relatives within our sample. Relatives were identified as those with a PI-HAT (proportion of IBD) threshold of greater than 0.1875; where 0.5 represents first-degree relatives and 0.25 represents second-degree relatives. Only a single member from each family were retained post quality control. Following quality control, the sample consisted of 351 unrelated individuals, for which we had both genome-wide genotype data and telomere length data.

### Polygenic Risk Score Quantification

#### PRSice Software

Individualized Polygenic Risk Scores within our sample were calculated using PRSice, a PRS quantification software (Euesden et al., 2015). The software uses summary results from previously performed, well-powered GWAS (the base dataset) to generate PRSs in our sample, SELCoH (the target dataset). Briefly, PRSice works by first clumping SNPs in the genotype PLINK files corresponding to the target dataset and removing those in high linkage disequilibrium, as this can falsely inflate polygenic scores. Subsequently, within the target dataset the number of risk alleles at a particular SNP is multiplied by that SNP’s effect size (established in the base dataset), and then all the SNP information is summed. The user can define which SNPs to include in the PRS. For all analyses we set a *p*-value threshold of *p* = 0.1, whereby we included all SNPs under this threshold from our base datasets to calculate polygenic risk scores in our target dataset.

#### Base Datasets

The MDD base dataset (summary statistics) was obtained from the Psychiatric Genomics Consortium (PGC) and represents the largest GWAS for depression to-date, consisting of 135,458 MDD cases and 344,901 controls (Wray et al., 2018). The base dataset for BD consists of GWAS results for 7,481 cases and 9,250 controls (Sklar et al., 2011). For the BD GWAS, SNP positions were lifted over from hg18 to hg19 build using UCSC LiftOver tool (Kuhn et al., 2013). The base dataset for SCZ consists of insights from a multi-stage schizophrenia genome-wide association study of up to 36,989 cases and 113,075 controls (Ripke et al., 2014). All base datasets were downloaded from the PGC website^1^.

#### Population Covariates

To reduce noise in our analyses as a result of ancestry differences within the sample, we generated population covariates (PCs) using multidimensional scaling in PLINK, via the PRSice software, which allowed us to detect and adjust for population structure in our analyses (Patterson et al., 2006; Price et al., 2006). Population covariates were incrementally tested for association via scatter charts (e.g., PC1 vs. PS2, PC2 vs. PC3, PC3 vs. PC4, etc.) until a normal distribution was achieved. A normal distribution was achieved after the first seven PCs, and thus this is what we used in our downstream analyses.

### Statistical Analysis

#### RTL Calculation

A standard deviation of less than 0.5 was required for at least two of the three cycle threshold (C*_t_*) technical triplicates for a sample to be included in downstream analysis. *C_q_* values were then created from the remaining *C_t_* values by relating them to absolute quantities as part of a standard curve. RTL was then calculated by dividing each sample’s mean *C_q_* value from the telomere reaction by each sample’s mean *C_q_* value from the albumin reaction. RTL was then log-transformed to allow for parametric analysis. Outliers were identified as those data points greater than two standard deviations from the mean and subsequently removed. As a final check, we performed a one-tailed Pearson correlation test to confirm there was a negative correlation between log(RTL) and age.

#### PRS and its Relationship to Telomere Length (Dep-Sample Only)

To determine the effect of each PRS on telomere length we performed three independent linear regressions, whereby log(RTL) was selected as our outcome; age, BMI, PCs 1-7, plate batch, smoking status (former, current, never), gender, and ethnicity were selected as covariates; and PRS was selected as our independent variable.

#### Antidepressant Use and Telomere Length (Full Sample)

The relationship between antidepressant use and telomere length was investigated using a linear regression, whereby log-telomere length was selected as the outcome variable; age, BMI, PCs 1-7, plate batch, smoking status (former, current, never), gender, ethnicity, depression diagnosis were included as covariates; and antidepressant use was used as our independent variable.

#### Antidepressant Use and Aging-Related Disease (Full Sample)

An ordinal logistic regression was used to determine the relationship between antidepressant use and the number of aging-related diseases (0/1/2). Number of aging-related diseases was selected as our outcome variable; age, BMI, PCs 1-7, smoking status (former, current, never), gender, ethnicity, and lifetime depression diagnosis were selected as covariates; with antidepressant use selected as the independent variable.

#### Sensitivity Analyses

We performed a series of sensitivity analyses to determine the relationship between telomere length and physical illnesses/medication use, and the potential confounding effects of depression severity.

#### Power Calculation

Power calculations indicate we have 100% power to detect small-moderate effect sizes (effect size = 0.3) for analyses (i) to (iii), given our sample size, with an α = 0.05.

## Results

### Quality Control Checks

Standard curves from all reactions showed an *R^2^* ≥ 0.98 between quantity of known DNA and *C_t_* values. Negative controls showed no amplification on any of the plates and a single peak was detected for the dissociation curves (melting curves) across all plates, demonstrating that binding specificity of the primers to the DNA was achieved to a high degree, see **Supplementary Information**. The telomere reaction achieved a mean efficiency of 90% and the albumin reaction achieved a mean efficiency of 79%. Efficiencies were all corrected via a standard curve on each of the plates and all samples which didn’t pass our quality control criteria were removed from any further analyses (17 samples).

### Telomere Length and Chronological Age

**FIGURE 1:**
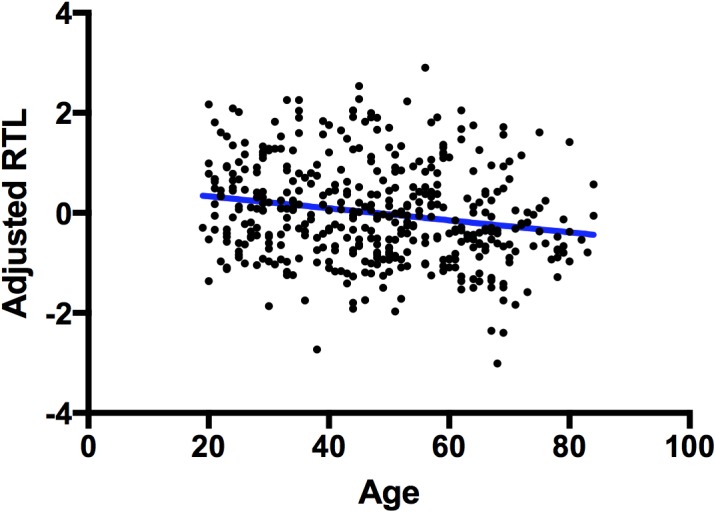
Correlation between telomere length and chronological age. A scatterplot showing a significant negative correlation between chronological age (*x*-axis) and adjusted RTL (*y*-axis). The blue line represents a line of best fit.

A one-tailed Pearson’s correlation showed that relative telomere length (adjusted for inter-plate variability) is negatively correlated with age in our whole sample, *r*(351) = -0.223, p 1.20E-05 (**Figure 1**) as expected.

### Polygenic Risk for Psychiatric Disorders and Telomere Length

**FIGURE 2:**
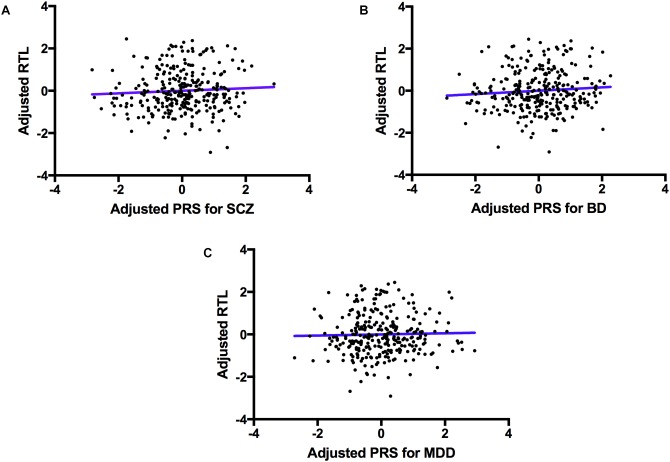
The association between polygenic risk for psychiatric disorders and telomere length. A scatter plot showing PRSs for SCZ (**A**, top left), BD (**B**, top right) and MDD (**C**, bottom) adjusted for PCs 1–7 (*x*-axis), and log(RTL) adjusted for age, sex, ethnicity, BMI, smoking status and telomere plate batch (*y*-axis). The blue line represents the line of best fit.

The regression model examining the effect of polygenic risk for SCZ on log(RTL) did not reveal a significant association (*F*(1,265) = 1.622, *p* = 0.204). Similarly, we did not find a significant association between the polygenic risk for BD and log(RTL; *F*(1,265) = 1.872, *P* = 0.172), nor between polygenic risk for MDD and log(RTL; *F*(1,265) = 0.519, *P* = 0.472), see **Figure 2**.

### The Effect of Antidepressants Use on Telomere Length

**FIGURE 3:**
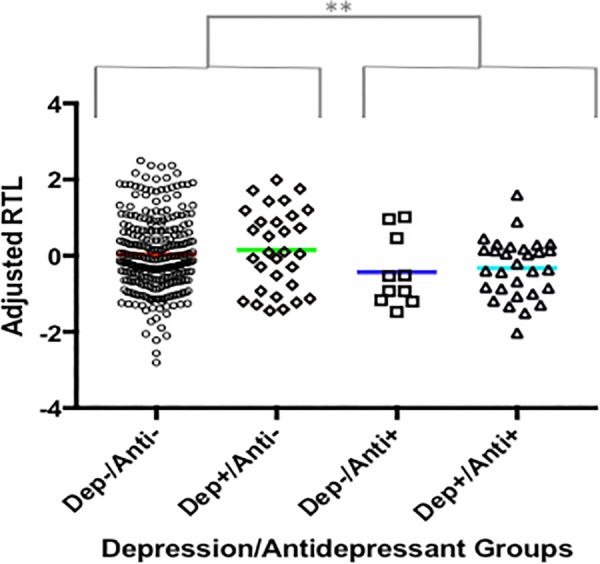
The association between antidepressant use and RTL. A plot showing adjusted RTL in participants: (i) without depression and who are not currently taking antidepressants (Dep-/Anti-), (ii) with a depression diagnosis and who are not currently taking antidepressants (Dep+/Anti-), (iii) without depression who are currently taking antidepressants, (iv) with depression who are currently taking antidepressants. Participants currently taking antidepressants had significantly shorter RTL compared to those not taking antidepressants, irrespective of depression diagnosis. The symbol ‘^∗∗^’ indicates a difference of *p* ≤ 0.01.

Antidepressant use was significantly associated with telomere length in our total sample, which was irrespective of depression diagnosis (*F*(1,325) = 6.575, *P* = 0.011, variance explained = 2%), see **Figure 3**.

### Antidepressant Use and Aging Related Disease

**FIGURE 4:**
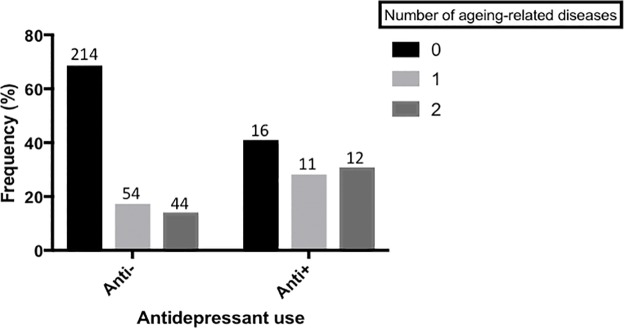
Antidepressant use and aging-related disease. A bar chart showing the frequency of participants with 0, 1 or 2(+) aging-related diseases, split by those who are not currently taking antidepressants and those who are. Actual sample size within each group is shown on top of each bar. There is a significant difference in the frequency of aging-related disease between the two groups (*p* < 0.05). Aging-related diseases included: type-2 diabetes, arthritis, cardiovascular disease, stroke, high blood pressure, and cancer.

We found that those currently taking antidepressants also had a higher frequency of aging-related disease, relative to those not currently taking an antidepressant. (Estimate = -0.981 (95% *C.I.* = -1.878, -0.084), *p* = 0.032), see **Figure 4**.

### Sensitivity Analyses

#### Telomere length and Aging-Related Disease

We performed ordinal logistic regressions to determine if log(RTL) predicts number of aging-related diseases (0,1,2) whilst covarying for age, BMI, PCs 1-7, smoking status (former, current, never), gender, ethnicity, lifetime depression diagnosis and antidepressant use. Log(RTL) did not predict number of ageing-related diseases (*p* > 0.05).

#### Telomere Length, Medication Use and Disease

We performed linear regressions which included age, sex, ethnicity and BMI as covariates, and log(RTL) as the outcome variable, alongside self-reported disease or medication use. No diseases or medications predicted log(RTL), *p* > 0.05, see **Supplementary Information** for further details.

#### Telomere Length, Antidepressant Use and Aging-Related Disease

We further tested whether telomere length mediated the association between antidepressant use and risk for aging-related disease. So we repeated analysis (iv) as described above: An ordinal logistic regression was used to determine the relationship between antidepressant use and the number of aging-related diseases (0/1/2 +; outcome variable). However, we also included log(RTL) as a covariate. The relationship between antidepressant use and number of aging-related diseases remained significant (*p* < 0.05), suggesting the effect was not mediated by telomere length.

#### Depression Severity at Blood Collection, and Telomere Length

To confirm depression severity did not confound analyses investigating the effects of antidepressants on log(RTL), we repeated analysis (iii), however, we further included depression severity (0 = none, 1 = mild, 2 = moderate/severe) as a covariate, and found that antidepressant use still predicted log(RTL; *p* < 0.05), suggesting episode severity was not confounding our result.

## Discussion

Previous studies have revealed higher rates of aging-related diseases amongst psychiatric disorder patients, with some studies indicating that shorter telomere length (and faster aging) may be the cause (Hippisley-Cox et al., 2001; Gareri et al., 2002; Caughey et al., 2010). The first aim of our study was to clarify whether genetic risk for psychiatric disorders also carries risk for shorter telomere length. To achieve this aim we generated PRS for MDD, BD, and SCZ, and assessed the relationship between these PRS and telomere length measurements in a cohort of individuals with no history of psychiatric health problems. We found no evidence to suggest that genetic risk for psychiatric disorders also contributes to telomere shortening, **Figure 2**. In terms of translational medicine, our results suggest that although polygenic risk scoring may be useful in predicting those at risk for psychiatric disorders, current psychiatric polygenic risk scores alone may not be useful in predicting those who are also susceptible to shorter telomeres and aging-related diseases. Instead, our results support previous work indicating the importance of environmental factors associated with psychiatric diagnosis, in accelerating telomere shortening (Kessler et al., 2005; Whiteford et al., 2013).

Indeed, we found evidence that antidepressant use was associated with shorter telomere length, an effect which was independent of depression diagnosis. This partially corroborates previous reports which found that only depressed patients currently taking antidepressants have shorter telomeres (Lindqvist et al., 2015; Needham et al., 2015). However, in contrast to previous studies which have suggested that antidepressant use is a proxy for current depression severity, and that this is what drives the association with shorter telomeres, our results show that even non-depressed participants who are taking antidepressants for other purposes (e.g., sleep) had similarly short telomere lengths, **Figure 3**. We further confirmed that current depression severity did not differ between depressed patients who were and were not taking antidepressants at the time of blood collection, suggesting the effect was not driven by current depression severity. One possible explanation is that antidepressants are increasing the proliferation of blood cells in users, and the knock-on effect is telomere shortening. Indeed, research using lymphoblastoid cell lines (resembling white blood cells), hippocampal progenitor cell lines, and *in vivo* studies of the hippocampus, support the notion that antidepressants increase proliferation (Manev et al., 2001; Breitfeld et al., 2017; Powell et al., 2017b). However, some work indicates that antidepressants may additionally increase the activity of telomerase, which is an enzyme involved in telomere maintenance and elongation (Bersani et al., 2015), therefore more work is needed to better understand the effects of antidepressants on telomere length and proliferation over time.

Interestingly, we additionally found an association between antidepressant use and risk for aging-related disease; with antidepressant use predicting a higher number of aging-related diseases, an effect which was independent of depression case/control status, **Figure 4**. This matches recent epidemiological reports that antidepressant use is associated with an increased risk for aging-related disorders such as cardiovascular disease (Musselman et al., 1998; Lichtman et al., 2008; Hamer et al., 2011). Interestingly, our sensitivity analyses suggest that the relationship between antidepressant use and aging-related disease is not mediated by telomere length variability, such that antidepressant use is independently associated with both risk for aging-related disease and shorter telomere length. These results indicate one of two things. First, that antidepressants increase telomere shortening and risk for aging-related disease via independent mechanisms. Second, and more likely, that antidepressant use, or prescription, is more common amongst those who suffer from depression (or related conditions such as sleep problems), and who also suffer from chronic debilitating aging-related disease. Nevertheless, the combination of previously reported epidemiological data linking antidepressant use with aging-related disease, and molecular data reported here, warrants further consideration of the long-term impact of antidepressant use on aging-related phenotypes. In particular, studies recruiting antidepressant users (both depressed and non-depressed) as part of a longitudinal design may help in discerning causality.

The main limitations of our study are the small number of individuals taking antidepressants, and the fact we may be underpowered to detect small effect sizes. Nevertheless, our study is the first to suggest that: (i) genetic risk for psychiatric disorders does not predict faster biological aging, (ii) antidepressant use is associated with shorter telomeres independently of depression diagnosis, (iii) antidepressant use is associated with an increased number of aging-related diseases, independently of depression diagnosis. Our work suggests that the relationship between antidepressant use and risk for aging-related disease may need to be reconsidered.

## Conclusion

We found no evidence to suggest that genetic risk for psychiatric disorders also contribute to faster telomere shortening, highlighting the potential importance of environmental factors in mediating physical disease comorbidity. We did, however, find an association between antidepressant use and telomere length, with antidepressant use being associated with shorter telomere length. In addition, we found antidepressant use to be associated with a higher number of aging-related diseases in participants, replicating previous epidemiological evidence. Further work is now needed to test whether antidepressants induce telomere shortening via their proliferative effects, and how antidepressant use relates to aging-related disease.

## Author Contributions

GB, LG, SF, SH, and MH did the sample collection, interpretation of results, final approval for publication. CL and ST interpreted the results and provided the final approval for publication. AP and TP created and designed the work, acquired the data, analyzed and interpreted the data, prepared and revised the manuscript, and provided the final approval for publication.

## Conflict of Interest Statement

GB has received grant moneyand acted as a consultant for Eli Lilly. The remaining authors declare that the research was conducted in the absence of any commercial or financial relationships that could be construed as a potential conflict of interest. The handling Editor declared a shared affiliation, though no other collaboration, with the authors.

## Footnotes

1 http://www.med.unc.edu/pgc/results-and-downloads/results
