# Supplementary material for: Genetic Risk for Psychiatric Disorders and Telomere Length
Source: Front Genet. 2018 Oct 16;9:468. doi: 10.3389/fgene.2018.00468 (PMC6232668; doi:10.3389/fgene.2018.00468)
Supplement: Supplementary file 1 [file Data_Sheet_1.DOCX]

**Telomere length and other traits:** All results are based on univariate linear regressions which included age, sex, ethnicity and BMI as covariates, and log(RTL) as the outcome variable.

| **Smoking and Drugs** | **F** | **d.f.** | **p-values** | **Effect Size** |
| --- | --- | --- | --- | --- |
| Smoking Status | .918 | 1 | .400 | .004 |
| Drug Dependency | .034 | 1 | .855 | .000 |
| Drug Use | 3.552 | 1 | .060 | .008 |

| **Illnesses** | **F** | **d.f.** | **p-values** | **Effect Size** |
| --- | --- | --- | --- | --- |
| Asthma | .013 | 1 | .909 | .000 |
| Depression/other Nervous Illnesses | .336 | 1 | .563 | .001 |
| Diabetes | .533 | 1 | .466 | .001 |
| Stomach/Digestive Disorders | .179 | 1 | .672 | .000 |
| Rheumatic Disorders/Arthritis | .513 | 1 | .474 | .001 |
| Heart Trouble | .450 | 1 | .503 | .001 |
| Stroke | .015 | 1 | .903 | .000 |
| High Blood Pressure | .195 | 1 | .659 | .000 |
| Migraines | .064 | 1 | .801 | .000 |
| Epilepsy | 2.970 | 1 | .086 | .007 |
| Gynaecological Problems | .210 | 1 | .647 | .001 |
| Cancer | .666 | 1 | .415 | .002 |
| Kidney Problems | 2.171 | 1 | .141 | .005 |
| Other | 1.942 | 1 | .164 | .005 |
| Long Lasting Illnesses | .135 | 1 | .713 | .000 |
| Number of Long Lasting Illnesses | .286 | 1 | .593 | .001 |

| **Medications** | **F** | **d.f.** | **p-values** | **Effect Size** |
| --- | --- | --- | --- | --- |
| Pain medication | .067 | 1 | .796 | .000 |
| Antacid Medication | .662 | 1 | .416 | .002 |
| Cold Medication | .232 | 1 | .630 | .001 |
| Allergy Medication | .636 | 1 | .426 | .002 |
| Antibiotic Medication | .001 | 1 | .973 | .000 |
| Birth Control Medication | .006 | 1 | .940 | .000 |
| Chest Medication | .057 | 1 | .811 | .000 |
| Diabetes Medication | .007 | 1 | .933 | .000 |
| Heart/blood pressure Medication | .296 | 1 | .587 | .001 |
| Thyroid Medication | .140 | 1 | .709 | .000 |
| Other Medication | .150 | 1 | .699 | .000 |
| Vitamin Supplements | .098 | 1 | .754 | .000 |
| Herbal Medication | 1.139 | 1 | .286 | .003 |
| Any Medication | .417 | 1 | .519 | .001 |
