# Supplementary material for: Genetic Risk for Psychiatric Disorders and Telomere Length
Source: Front Genet. 2018 Oct 16;9:468. doi: 10.3389/fgene.2018.00468 (PMC6232668; doi:10.3389/fgene.2018.00468)
Supplement: Supplementary file 2 [file Image_1.pdf]

MELTING CURVES

Telomere reaction

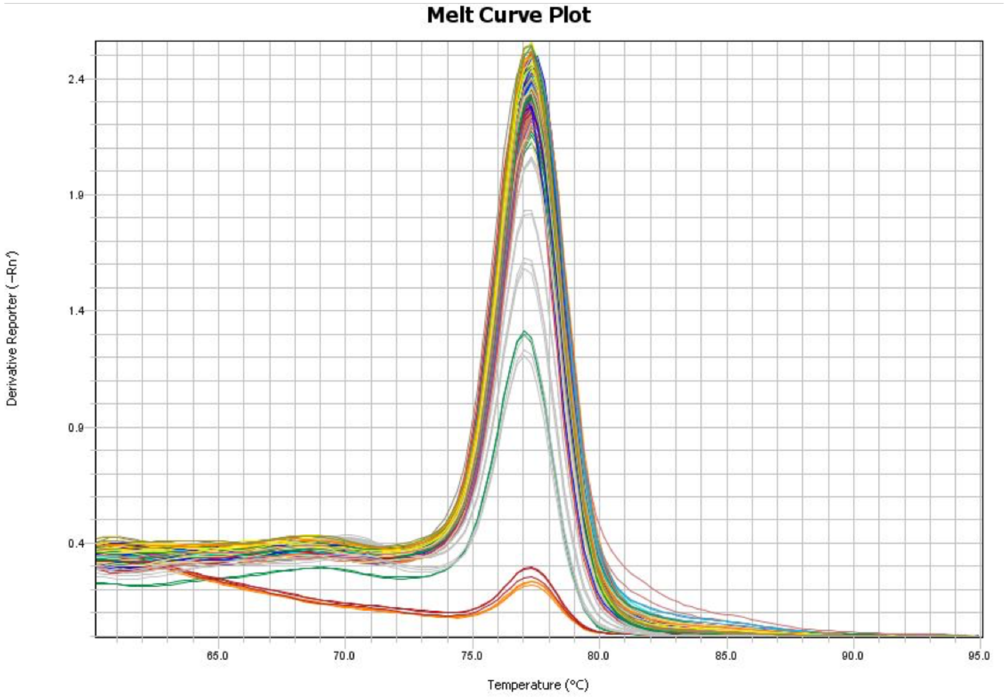

Albumin reaction

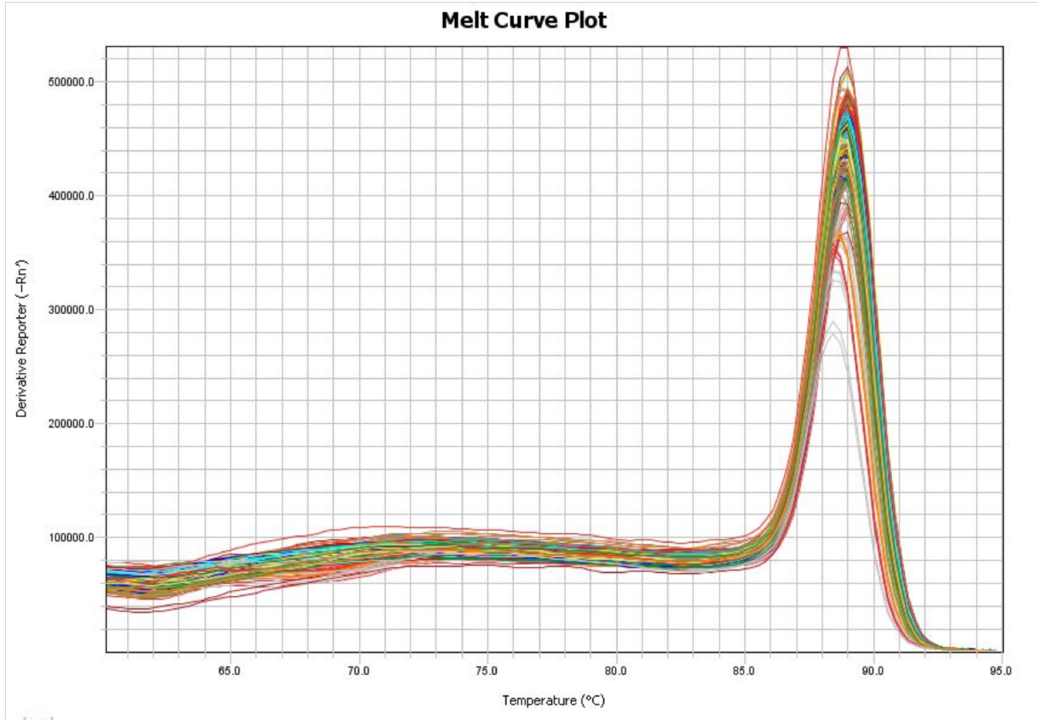

Note: For both reactions there was a single clear peak indicating the absence of non-specific amplification.
